# Supplementary material for: High Detection Frequency of Vaccine-Associated Polioviruses and Non-Polio Enteroviruses in the Stools of Asymptomatic Infants from the Free State Province, South Africa
Source: Microorganisms. 2024 Apr 30;12(5):920. doi: 10.3390/microorganisms12050920 (PMC11124149; doi:10.3390/microorganisms12050920)
Supplement: Supplementary file 1 [file microorganisms-12-00920-s001.zip › microorganisms-2878265-supplementary.pdf]

Supplementary material S1

Genome coverage maps of enterovirus B subtypes

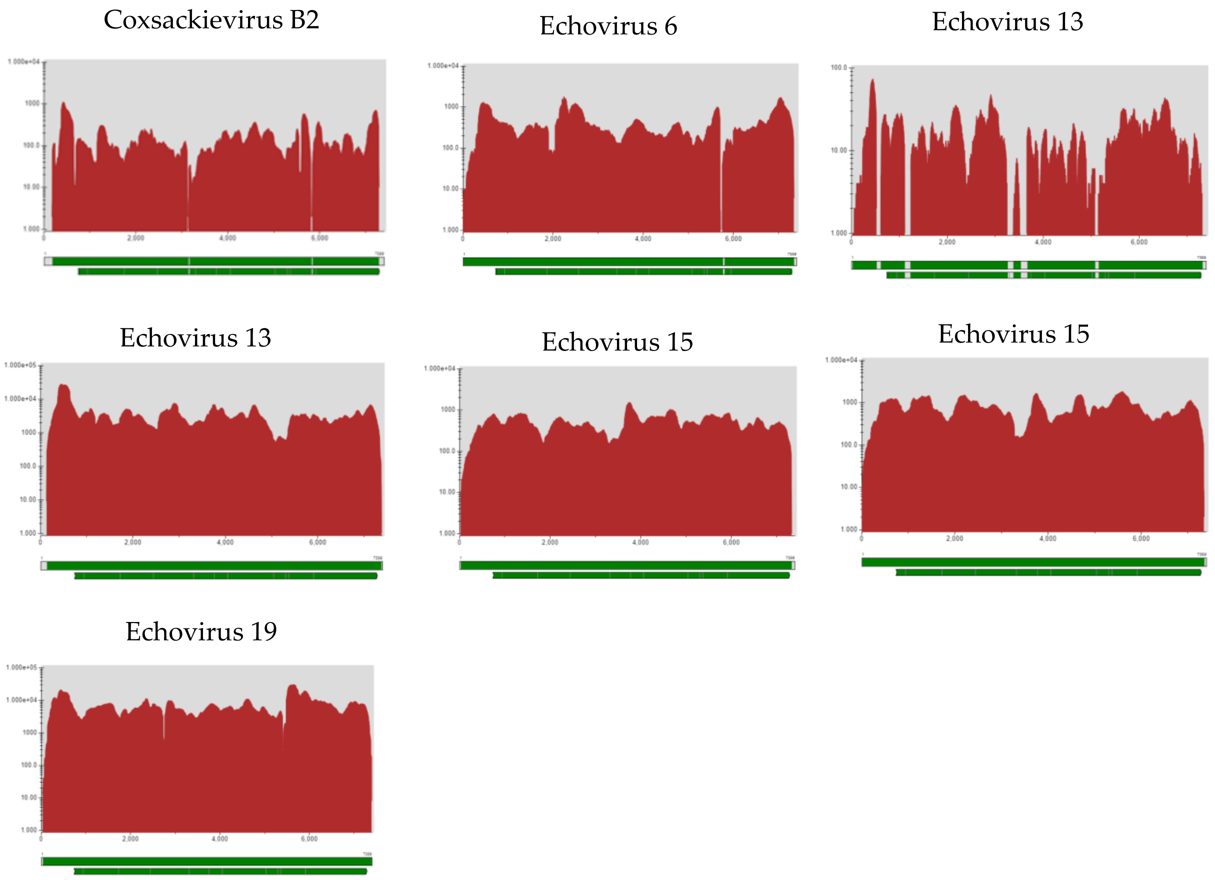

Figure S1: *Genome coverage maps of the seven enterovirus B subtypes identified (coxsackievirus B2, echovirus 6, 13, 15, and 19).*

Genome coverage maps of enterovirus C subtypes

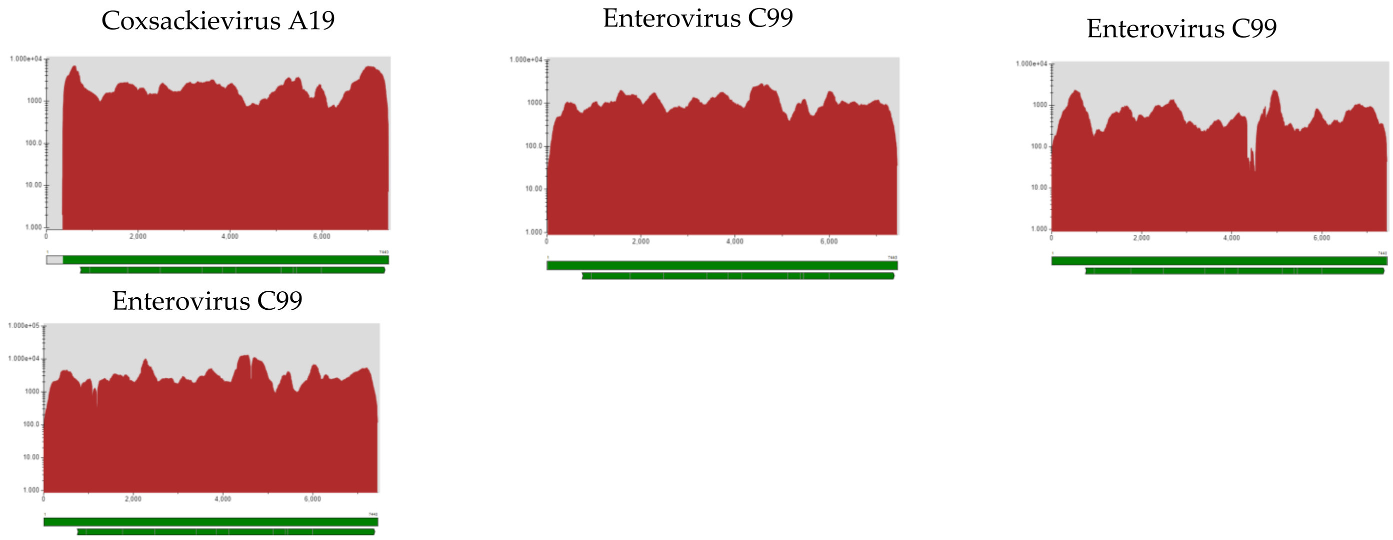

Figure S2*: Genome coverage maps of the seven enterovirus C subtypes identified (coxsackievirus A19, Enterovirus C99).*
